# Myocardial Dysfunction in Acute Traumatic Brain Injury Relieved by Surgical Decompression

**DOI:** 10.1155/2013/482596

**Published:** 2013-06-04

**Authors:** Vijay Krishnamoorthy, Deepak Sharma, Sumidtra Prathep, Monica S. Vavilala

**Affiliations:** ^1^Department of Anesthesiology and Pain Medicine, University of Washington, WA 98104, USA; ^2^Departments of Anesthesiology and Pain Medicine, Neurological Surgery (Adj.), University of Washington, WA 98104, USA; ^3^Departments of Anesthesiology and Pain Medicine, Neurological Surgery (Adj.), Pediatrics (Adj.), and Radiology (Adj.), University of Washington, WA 98104, USA

## Abstract

Traumatic brain injury (TBI) is a major public health issue and is a leading cause of death in North America. After a primary TBI, secondary brain insults can predispose patients to a worse outcome. One of the earliest secondary insults encountered during the perioperative period is hypotension, which has been directly linked to both mortality and poor disposition after TBI. Despite this, it has been shown that hypotension commonly occurs during surgery for TBI. We present a case of intraoperative hypotension during surgery for TBI, where the use of transthoracic echocardiography had significant diagnostic and therapeutic implications for the management of our patient. We then discuss the issue of cardiac dysfunction after brain injury and the implications that echocardiography may have in the management of this vulnerable patient population.

## 1. Introduction

 Traumatic brain injury (TBI) is a major public health issue and is a leading cause of death in North America [1]. After a primary TBI, the burden of secondary brain insults can predispose patients to a worse outcome than if secondary insults did not occur [2, 3]. One of the earliest secondary insults encountered during the perioperative period is hypotension, which has been directly linked to both mortality and poor disposition after TBI [4, 5]. While recommendations of the 2007 Brain Trauma Foundation recommend maintaining systolic blood pressure (SBP) >90 mmHg [6], it has recently been shown that reduction in SBP to values below 90 mmHg commonly occurs during surgery for TBI. Risk factors for intraoperative hypotension include large lesions and the presence of multiple lesions on CT [7]. Therapy for intraoperative hypotension has traditionally consisted of the administration of intravenous fluids and vasopressors, and vasopressor choice in this setting is often empiric. There are no guidelines specific for treatment of hypotension during the intraoperative period; thus, knowledge of a patient's preexisting cardiac status may impact anesthesiologists' choice of vasopressor for treatment of intraoperative hypotension.

In this report, we present the clinical course of a patient with a traumatic holohemispheric subdural hematoma (SDH), where echocardiographic changes consistent with myocardial dysfunction were observed upon admission to the operating room. The echocardiographic abnormalities were rapidly reversed after craniotomy and surgical decompression. In this case, the use of point of care (POC) intraoperative transthoracic echocardiography (TTE) allowed for timely identification of a cardiac cause of hypotension, and it facilitated appropriate vasopressor choice for treatment. In addition, the phenomenon of reversible cardiac dysfunction after traumatic intracranial hemorrhage, relieved by decompression, is postulated. IRB approval was not required for submission of this case.

## 2. Case Presentation

**Figure 1:**
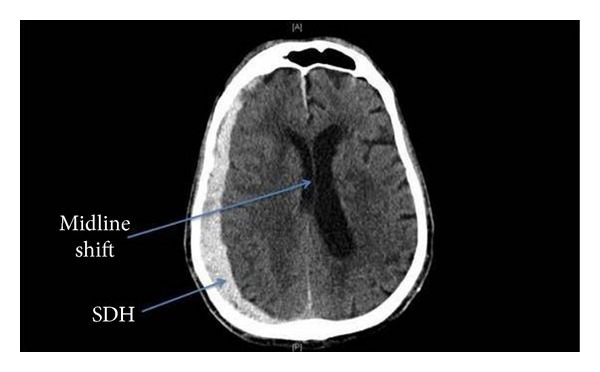
Right holohemispheric subdural hematoma (SDH) and midline shift in a 76-year-old male, resulting in neurologic deterioration and emergent surgical decompression management.

The patient was a 76-year-old functionally independent male with a preexisting history remarkable only for hypertension and bipolar disorder. On review of systems, he was found to have a negative cardiac history and no treatment with anticoagulants. He presented to an outside hospital with declining mental status. The patient experienced a ground-level fall and worsening somnolence. At the outside hospital, computed tomography (CT) scan of his head demonstrated a right holohemispheric subdural hematoma (SDH), measuring 1.7 cm at maximal thickness, and with an associated mild 5 mm midline shift (Figure 1). Due to his worsening mental status, the patient's trachea was intubated, and he was transferred to our hospital (regional Level 1 Trauma Center), where he underwent an emergent right craniotomy and SDH evacuation. The patient tolerated the procedure well, and his trachea was extubated at the end of surgery. 

**Figure 2:**
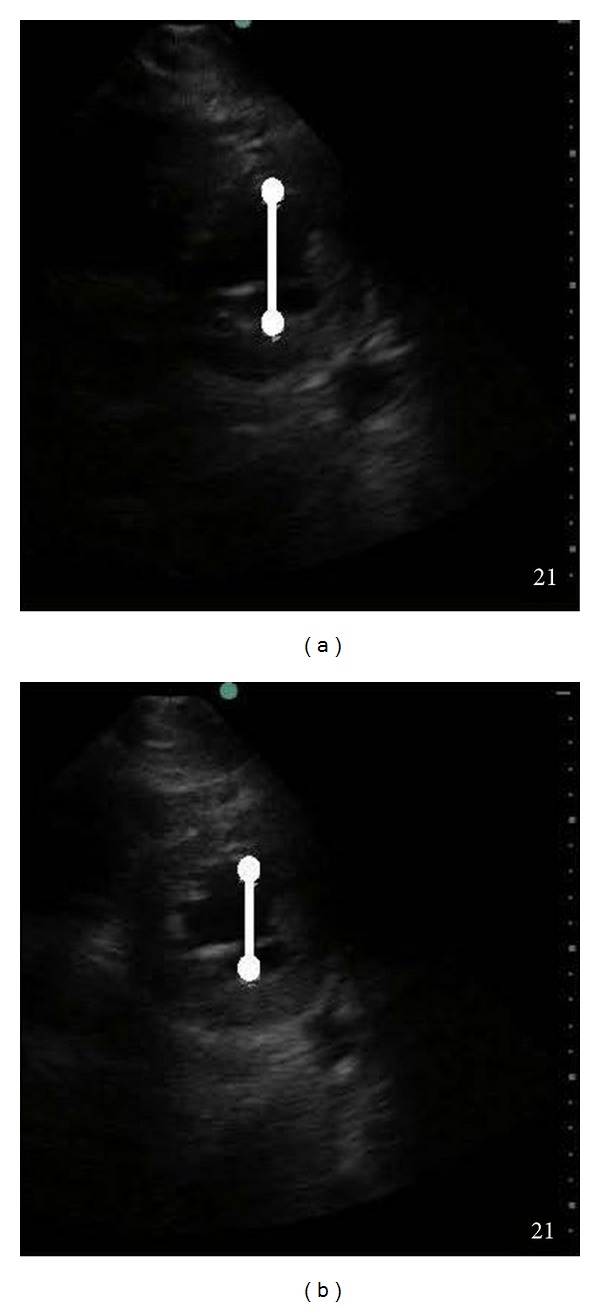
Left-ventricular function at end-systole pre decompression (a) and post decompression (b). There is decreased left-ventricular end-systolic internal diameter (line) and improved wall thickening after decompression.

 On hospital day two, he again became increasingly somnolent and had generalized seizure activity; a stat head CT scan revealed a new acute right-sided subdural hematoma in the same area as the previous traumatic lesion with a new 6 mm midline shift and uncal herniation. Laboratory values were significant for a hematocrit of 35% and an international normalized ratio (INR) of 1.2; and his vital signs were significant for a blood pressure of 140/92 mmHg and a heart rate of 112 bpm. The patient was emergently brought to the operating room for repeat surgical evacuation and hemicraniectomy. General anesthesia was induced with 100 mg propofol, 100 mcg fentanyl, and 100 mg rocuronium administered intravenously. The patient underwent a rapid sequence tracheal intubation with cricoid pressure, and general anesthesia was maintained with intravenous boluses of fentanyl, vecuronium, and isoflurane. After induction of general anesthesia, the patient's systolic blood pressure decreased to 54 mmHg. Multiple intravenous boluses of phenylephrine (totaling 300 mcg) and 750 mL of crystalloid were administered with only minimal improvement and with persistent hypotension (systolic blood pressures less than 90 mmHg). Intraoperative POC TTE was performed by an anesthesiologist with certification in echocardiography: Vijay Krishnamoorthy, which demonstrated a global ejection fraction (EF) of 35% and moderate basal hypokinesis (Figure 2(a)). Based on these echocardiographic findings, the need for an intravenous inotropic vasopressor was identified, and due to ease of availability, 20 mg intravenous ephedrine was administered over five minutes with sustained improvement in systolic blood pressure to greater than 120 mmHg during the next 10 minutes without the need for additional vasopressor support or intravenous fluids. While improvements in EF were noted on TTE after pharmacologic therapy, the EF had not returned to an expected normal level. 

Five minutes after surgical decompression of the SDH, a third POC TTE was performed, this time revealing resolution of the previously observed basal hypokinesis and normalization of the EF to 55% (Figure 2(b)). The systolic blood pressure remained above 100 mmHg without the need for any further vasopressor support (Table 1). The remainder of the case proceeded uneventfully, and the patient remained intubated and was transferred to the ICU in stable condition. After a 25-day hospital course, the patient was discharged to a skilled nursing facility for further care.

## 3. Discussion

While intraoperative hypotension during acute decompressive surgery for TBI has previously been described, the etiology of hypotension has not been clearly elucidated. Hence, the traditional approach to intraoperative hypotension during TBI surgery has been empiric and assumed to be a result of the acute cardiopulmonary physiologic stress of brain injury [8], fluid shifts, and effects of anesthetic agents [9, 10]. The therapeutic approach to the treatment of hypotension during decompressive surgery for TBI has involved the administration of intravenous fluids to restore euvolemia, followed by intravenous vasopressors which have alpha-adrenergic effects and those without cerebral effects [11]. In TBI, intravenous phenylephrine has been reported to be the most frequently administered intraoperative vasopressor [12]. Despite this practice, available evidence suggests that vasopressors with inotropic effects such as norepinephrine might be preferable as the vasopressor of choice in acute neurologic injury [13]. This case documents cardiac dysfunction acutely after TBI and suggests that anesthesiologists should consider point of care evaluation of cardiac function in their decision of vasopressor choice acutely after TBI.

The idea that acute neurologic injury causes acute cardiopulmonary dysfunction is not new. A variety of reports have documented electrocardiographic changes and elevations of cardiac-specific biomarkers in the setting of acute brain injury [14–16], with echocardiographic wall motion abnormalities being observed in patients with subarachnoid hemorrhage [17]. In addition, transient left-ventricular dysfunction (traditionally including apical ballooning) without the presence of preexisting coronary artery disease has been well described after subarachnoid hemorrhage, acute physical stress, or psychological stress, with overactivity of the sympathetic nervous system implicated in the pathogenesis [18, 19]. While these reports suggest that brain-heart interaction may also cause myocardial dysfunction in TBI, direct echocardiographic changes in the perioperative period after TBI have not been previously documented, nor has echocardiographic resolution of myocardial dysfunction following decompression been previously described in the literature. In our case, the critical determinant for improvement in the patient's cardiac function was surgical decompression.

The underlying mechanism of acute cardiopulmonary deterioration secondary to TBI is thought to be secondary to an acute catecholamine excess state [20], with recent evidence suggesting that inflammation may also play a role [21]. This idea is based on the speculation that increased intracranial pressure can cause increased central catecholamine outflow, resulting in both stress cardiomyopathy and isolated acute lung injury, better known as neurogenic pulmonary edema [22]. Interestingly, myocardial biopsies from brain-dead organ donors and survivors of stress-induced cardiomyopathy show mononuclear infiltrates and contraction-band necrosis, which indicate evidence of catecholamine-induced myocardial injury [18, 23]. 

 While stress-induced myocardial dysfunction can transiently decrease cardiac output, a secondary effect of acute heart failure is rapid accumulation of extravascular lung water, thus potentially aggravating hypoxia. Hypoxia has been shown to be an independent predictor of worsened outcomes in TBI, and the combination of hypotension and hypoxia leads to worse outcomes than either factor alone [24]. Thus, it is a possibility that treating hypotension secondary to acute heart failure empirically with intravenous fluids and pure alpha-agonists may lead to a vicious cycle of worsened blood pressure and increasing hypoxia. 

Traditionally in the perioperative setting, a craniotomy for a deteriorating patient with TBI is a medical emergency without the time for formal cardiac evaluation, often times leading to the treatment of hypotension without knowing the underlying etiology. While treating intraoperative hypotension is critical to avoiding secondary TBI and improving outcomes, treatment is often undertaken empirically. If the cause of intraoperative hypotension is acute myocardial stunning, perhaps the traditional therapy of fluids and increased afterload with phenylephrine may not be optimal. Instead, consideration of a vasopressor with inotropic activity and a conservative fluid strategy may better improve secondary hemodynamic goals to optimize outcome, especially since phenylephrine has been shown to decrease cardiac output [25], despite often being the vasopressor of choice in the setting of intraoperative management of TBI [12]. 

In conclusion, we report a case of intraoperative hypotension, where intraoperative POC TTE guided the hemodynamic management of a patient with TBI presenting for emergent decompressive craniotomy. This case highlights the growing role that POC ultrasound technology could add to the anesthesiologists' strategies for management of perioperative hemodynamic and respiratory instability. In addition, the observed cardiac dysfunction in our case likely represents a variant of “stress-induced” cardiomyopathy in TBI. Finally, to our knowledge, our case is the first to demonstrate the reversibility of cardiac dysfunction in TBI after surgical decompression, setting the stage for further research inquiry into this subject. 

## Figures and Tables

**Table 1:** Changes in qualitative ejection fraction (EF) and regional wall motion abnormalities (RWMA) pre and post decompressive craniotomy in a patient with isolated traumatic brain injury.

	Pre decompression	Post decompression
Qualitative EF	35%	55%
RWMA	Basal hypokinesis	None
Blood pressure	88/54 mmHg*	110/62 mmHg
Heart rate	84 bpm	92 bpm
Total crystalloid	1200 mL**	1600 mL
Hematocrit	35%	31%
Anesthesia	Sevoflurane	Sevoflurane
End-tidal sevoflurane	0.5%	1.5%

*: Ephedrine, 20 mg ephedrine administered to restore blood pressure to 124/72 mmHg.

**: Intravenous fluids administered over 1.5 hours, totaling 1600 mL post decompression (400 mL administered after surgical decompression).
